# Tailoring the Mass Density of 3D Printing Materials for Accurate X-ray Imaging Simulation by Controlled Underfilling for Radiographic Phantoms

**DOI:** 10.3390/polym16081116

**Published:** 2024-04-16

**Authors:** Ahmed Mahmoud Mabrouk Ahmed, Martin Buschmann, Lara Breyer, Claudia Kuntner, Peter Homolka

**Affiliations:** 1 Center for Medical Physics and Biomedical Engineering, Medical University of Vienna, 1090 Vienna, Austria; 2 Division of Medical Radiation Physics, Department of Radiation Oncology, Medical University of Vienna, and University Hospital Vienna, 1090 Vienna, Austria; martin.buschmann@akhwien.at; 3 Department of Biomedical Imaging and Image-Guided Therapy, Medical Imaging Cluster (MIC), Medical University of Vienna, 1090 Vienna, Austria; lara.breyer@meduniwien.ac.at (L.B.); claudia.kuntner@meduniwien.ac.at (C.K.)

**Keywords:** radiographic phantoms, additive manufacturing, 3D printing, medical imaging, X-ray imaging, computed tomography, tissue equivalent materials

## Abstract

Additive manufacturing and 3D printing allow for the design and rapid production of radiographic phantoms for X-ray imaging, including CT. These are used for numerous purposes, such as patient simulation, optimization of imaging procedures and dose levels, system evaluation and quality assurance. However, standard 3D printing polymers do not mimic X-ray attenuation properties of tissues like soft, adipose, lung or bone tissue, and standard materials like liquid water. The mass density of printing polymers—especially important in CT—is often inappropriate, i.e., mostly too high. Different methods can be applied to reduce mass density. This work examines reducing density by controlled underfilling either realized by using 3D printing materials expanded through foaming during heating in the printing process, or reducing polymer flow to introduce microscopic air-filled voids. The achievable density reduction depends on the base polymer used. When using foaming materials, density is controlled by the extrusion temperature, and ranges from 33 to 47% of the base polymer used, corresponding to a range of −650 to −394 HU in CT with 120 kV. Standard filaments (Nylon, modified PLA and modified ABS) allowed density reductions by 20 to 25%, covering HU values in CT from −260 to 77 (Nylon), −230 to −20 (ABS) and −81 to 143 (PLA). A standard chalk-filled PLA filament allowed reproduction of bone tissue in a wide range of bone mineral content resulting in CT numbers from 57 to 460 HU. Controlled underfilling allowed the production of radiographic phantom materials with continuously adjustable attenuation in a limited but appropriate range, allowing for the reproduction of X-ray attenuation properties of water, adipose, soft, lung, and bone tissue in an accurate, predictable and reproducible manner.

## 1. Introduction

In medical imaging, phantoms are important tools for exploring different research areas, and also have many applications in patient care. Phantoms are used as models for specific imaging tasks, in dosimetry or system evaluation [1,2]. They enable researchers to study a wide variety of research questions and improve patient care by optimizing their imaging procedures and treatments. Typically, a phantom acts as a physical or computerized model for a specific imaging, dosimetry or system evaluation task.

By employing realistic phantoms, diagnostic performance can be evaluated, imaging protocols tested and optimized, and radiation safety standards accessed across multiple imaging techniques. What sets a phantom apart from a test plate or object is that phantoms are designed to emulate the essential characteristics of patients with regard to the imaging modality and system settings (beam energy, e.g., in X-ray photon imaging).

Phantoms are usually custom-tailed for predefined applications, and can be very simple, like simple slabs [3,4] or stylized phantoms [5] just mimicking a typical patient attenuation in a medical X-ray field, or very complex. Anthropomorphic task-specific phantoms designed for studying the effects of, e.g., dose level variation, radiation quality, or system specifications on diagnostic performance, represent the most demanding category with regard to the realistic reproduction of relevant features [6,7,8,9,10,11,12]. Physical phantoms designed for dosimetry [13,14,15] need to closely duplicate the secondary radiation field, including long- and short-ranged secondary particles and photons, to allow for realistic dose measurements. Since, in many cases, this is difficult to achieve and extremely labor-intensive, mathematical phantoms and appropriate Monte Carlo simulation codes are very common in dosimetry applications [16,17] and image quality studies, including virtual trials [18,19]. Another application includes calibration [20,21] and accuracy test phantoms for quantitative imaging [21,22,23,24]. Phantoms for diagnostic image quality determination or optimization, on the other hand, are often combined with advanced image evaluation models like model observers, trying to forecast diagnostic performance of human observers like radiologists [25,26,27]. In these very demanding applications, it is crucial that the physical phantoms used realistically mimic the relevant patient properties of both healthy normal tissues and pathologies, with regard to structures [10,12,28], backgrounds [29,30,31], and X-ray interaction properties [32,33,34,35,36,37,38].

An additional complication in phantom design and productions is represented by phantoms where the haptics and mechanical properties of the tissue are important. This may even include non-linear viscoelastic properties of real soft tissues [39]. Applications include phantoms used for biopsy training [40], multimodal uses including X-ray and ultrasound, mammography, or surgical planning [41]. When soft tissues need to be mimicked with both appropriate radiation attenuation and mechanical properties, 3D-printable PVA hydrogels [42] or soft resins or filaments present a suitable option. To adjust the X-ray properties, these materials may need to be doped with appropriate additives.

If realistic structures are necessary in a phantom, additive manufacturing and 3D printing allow for realistic reproduction of anatomy or pathological patterns with a degree of fidelity and naturalism not achievable with other technologies, combined with a time-efficient design and production process. Therefore, additive manufacturing with 3D printing has become an indispensable technology in medical imaging [1,2,32,43].

To mimic liquid water (often used in phantoms) or body tissues for polychromatic X-ray photon spectra in an appropriate energy range, the effective atomic number of phantom material and material or tissue mimicked need to be similar, or at least close. However, most polymers used in 3D printing exhibit too low (effective) atomic numbers. This is seen in the energy dependence of X-ray attenuation. Quite extensive work has been performed identifying printing materials best mimicking water and tissue attenuation over a wider energy range in the keV region [32,44,45]. To simulate breast tissues in mammography at low keV photon energies, several SLA/DLP resins [46,47] and FDM polymers (Nylon and PET-G [46]) were found appropriate. At higher energies, as used in general radiography, fluoroscopy, interventional radiology, and CT, the most appropriate 3D-printable materials regarding attenuation and energy dependence were calcium carbonate and chalk-filled polymers to mimic bone tissues [45], and Nylon, ABS or ASA to mimic adipose tissue. However, all of these exhibit too high Hounsfield unit (HU) values in CT, corresponding to a too high mass density if printed with standard filament density. Reducing print density by 8 to 16% depending on the polymer would result in almost perfect tissue equivalence with, e.g., adipose tissue in the energy range of 70 to 140 kV X-ray spectra [45].

The mass density of printed samples can be controlled by different ways. For regular polymer filaments in FDM printing, infill patterns with reduced infill can be used [33,48,49,50], or the extrusion rate can be reduced during printing [28,34]. However, when reducing mass density by using infill patterns, these patterns will be visible in the final printed object as a regular periodic structure [49] that will compromise measurements in the spatial frequency domain or limit spatial resolution in the phantom. Another possibility is using filaments containing a heat-activated foaming agent, resulting in volume expansion in the heated printing nozzle. Several of these materials have become commercially available, normally based on PLA or ABS. These printing filaments are mainly aimed at the production of RC airplanes or aeromodelling parts, providing high structural stability combined with low mass. The resulting density is controlled by the nozzle temperature, where in most of the available materials, lower printing temperatures are associated with increased foaming; at higher temperatures, the produced gas will have a higher probability to escape from the now softer material before hardening, due to cooldown, resulting in less foaming and thus less expansion, leading to a higher mass density. The volume increase due to foaming is compensated for by reducing the flow rate to the point where neither under- nor over-extrusion occurs, i.e., where thin structures are printed with the correct dimensions. Optimum flow rates for all temperatures used need to be determined prior to printing the desired models by producing test samples and varying either the temperature or flow rate until dimensional accuracy is achieved.

The main aim of this work was to explore the density ranges possible with both standard and foaming polymer filaments, and to find the most suitable ones to produce tissue equivalent phantom materials for use in the keV photon range. This is most important for CT applications, since both mass and linear attenuation coefficients must mimic the tissues closely for all spectra/beam energies from 70 to 140 kV tube voltage. Samples printed were measured in a clinical scanner (Siemens Definition AS, Siemens Healthineers, Erlangen, Germany) to determine X-ray attenuation in Hounsfield units, and in a high-resolution Micro-CT (Siemens Inveon, Siemens Healthineers) to visualize the inner structure and homogeneity of the printed samples.

## 2. Materials and Methods

Standard commercially available (off-the-shelf) printing filaments were used in this study. To facilitate the time-efficient production of the test samples, three fused deposition modeling (FDM) printers were used simultaneously, comprising an Ultimaker S3 (Ultimaker BV, Utrecht, The Netherlands), an Anycubic Viper (Shenzhen Anycubic Technology Co., Ltd., Shenzhen, China) and an Flsun V400 (Zhengzhou Chaokuo Electronic Technology Co., Ltd., Zhengzhou, China). The filaments used and the associated printers are shown in Table 1.

The choice of filaments used was based on the filaments most suitable for radiographic phantoms [45]. Printing parameters used were either taken from [32] with adjustments if necessary, or orientated themselves on the specifications provided by the filament manufacturers. Layer heights of 0.1 mm were used for standard filaments, and 0.2 mm for foaming filaments. All printing was performed with 0.4 mm nozzles.

### 2.1. Density Reduction and Sample Production

For regular printing filaments (PLA, Nylon, ABS), the density reduction was realized by reducing the material flow rate in the printing process. However, it needs to be emphasized that, depending on the filament, flow rates above 100% commonly need to be used to produce printouts with the maximum achievable density [32]. In this work, flow rates down to 50% were explored in PLA to study the maximum possible reduction before the internal structure of the printed object collapses. In Nylon and ABS, the minimum flow rate used was 75%, since this rage covers all applications envisaged.

For foaming filaments, the rate of foaming is controlled by the extrusion temperature. While manufacturers provide guidance, calibration is advised for individual setups. The calibration procedures aim to find the relation between flow rate and printing temperature resulting in dimensionally correct printouts. Typically, an empty cube with a wall thickness of 2 times the nozzle size (0.8 mm for 0.4 mm nozzle orifice in this work) is printed, and the achieved wall thickness measured. If too thin, the flow rate is increased; if too thick, the flow rate is decreased. For every temperature, several flow rates slightly below and above the optimum one were used, and a linear regression was applied to find the value where the desired (accurate) thickness is reached. This procedure is repeated for all desired temperatures within the working range of the filament. In this work, 5 °C intervals were used. Minimal and maximal useable temperature is tested by extending the range until the filament either becomes practically unprintable, or shows signs of thermal damage or burning like darkening of the color.

Cylindrical samples with 2 cm diameter and 3 cm height were printed for determination of achieved densities and scanning in the clinical CT scanner. For high-resolution scanning using the Micro-CT scanner, smaller samples (2 cm diameter, 1.2 cm length) were produced to best exploit the limited scan field of view. Printing files in .stl format were generated in Fusion 360 (Autodesk Inc., San Francisco, CA, USA), and sliced in Cura (Ultimaker BV, Utrecht, The Netherlands, Version 5.3.0).

### 2.2. CT Scanning Procedures and Measurement of HU

**Figure 1:**
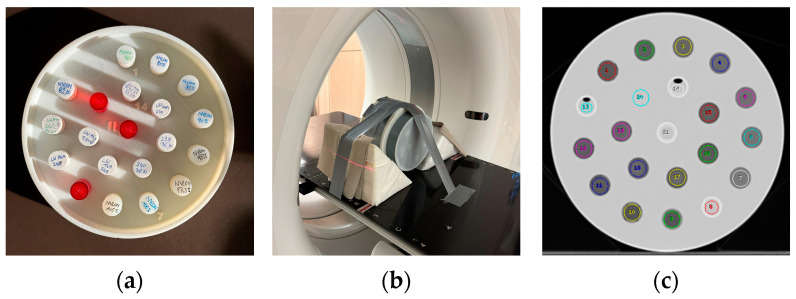
(**a**): Sample phantom section, (**b**): assembled phantom placed in medical CT scanner, and (**c**): sample slice with regions of interest used for measuring Hounsfield numbers.

For CT scanning in a medical full body scanner, the cylinders were embedded into phantom slices consisting of a 3D printed shell using the same PLA filament as specified in Table 1. The cylinders were fixed in the phantom using a mounting glue (Pattex Montagekleber white, Henkel, Düsseldorf, Germany). Phantom shells accommodated 13 to 21 cylinders. However, not all positions were filled in all phantom slices, and cylinders containing liquid water were added. Before mounting the phantom slices together, they were filled with a 7% *w*/*w* aqueous gelatin solution (Ballistic type 2, Gelita, Eberbach, Germany), closely mimicking water or soft tissue in the CT scanning process, to ensure appropriate scan conditions for standard reconstruction. Figure 1 shows a phantom section containing 21 cylinders and the assembled phantom positioned in the CT scanner. The red cylinders contain liquid water to check Hounsfield number accuracy and to measure noise in homogeneous media.

CT scanning to determine Hounsfield numbers was performed in a Siemens Somatom Definition AS (Siemens Healthineers, Erlangen, Germany) using a modified standard abdomen protocol applying 120 kV, 64 × 0.6 mm collimation, 2 mm reconstrued slice thickness and reconstruction increment, and a reconstruction kernel I40s (medium) with Safire strength 3. A lower pitch factor (0.5) in combination with 1 s rotation time was selected to allow high mAs (800) with lower tube loading to result in a low noise image stack.

**Figure 2:**
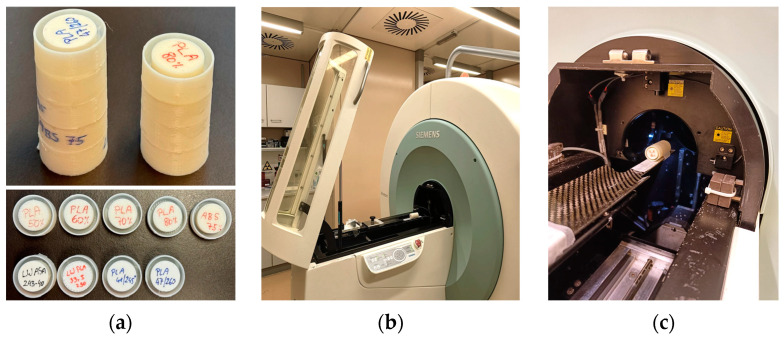
(**a**): Test samples printed for scanning in the Micro-CT scanner and assembled into 2 phantoms. (**b**,**c**): Micro-CT phantom loaded into the scanner.

In order to visualize the internal structure of the printed samples at the maximum achievable spatial resolution, a selected subset of samples was scanned in a Micro-CT scanner (Inveon Trimodality imaging system, Siemens Healthineers, Erlangen, Germany) [51]. This scanner is equipped with a variable focal spot X-ray source, a movable imaging bed, and a CCD camera as a detector. The imaging protocol employed 80 kV, 1 mm Al filter, 0.5 mA tube current, and 200 ms exposure time per projection. Data were acquired in step-and-shoot mode over 360 degrees using 1 projection per degree of rotation, with a settle time between the projections of 1 s. Raw data were reconstructed using a Feldkamp cone beam algorithm with an 800 × 800 × 1984 matrix, and a binning factor of 2, resulting in an isotropic voxel size of 35.421 µm. Due to the small field of view (28.34 mm diameter and 70.28 mm length) in the high-resolution mode, smaller samples with 2 cm diameter and 12 mm length were produced to allow a maximum of 5 samples to be scanned in one scan procedure. Each sample was glued into a 3D printed cup with a 2.5 cm diameter, and 5 and 4 samples, respectively, were glued together to form two phantoms, making the best use of the available scan length (Figure 2). For scanning in the Micro-CT scanner, samples of PLA and modified ABS printed with 50, 60, 70 and 80% flow rate (PLA), and with 75% flow rate (modified ABS) were used. From the lightweight (foaming) ASA and PLA, 4 cylinders each were produced to be scanned in the Micro-CT. For light-weight PLA, as the most promising foaming material, 3 densities were scanned comprising the minimum achievable density (34%), a medium (41%), and the maximum achievable density (47%). LW ASA was examined at a medium density (40%).

## 3. Results

### 3.1. Light-Weight Foaming Materials

**Figure 3:**
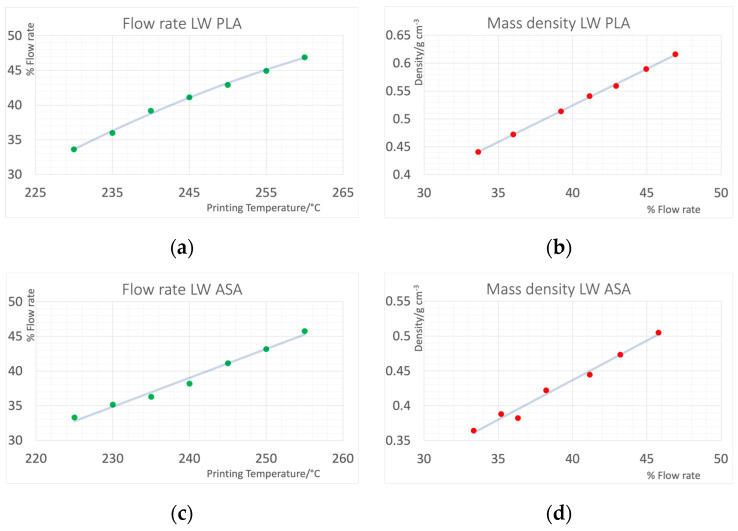
(**a**,**c**) Flow rates and (**b**,**d**) resulting mass densities for the two foaming filaments determined in a test print series. (**a**,**b**) Light-weight (LW) PLA, (**c**,**d**) Light-weight (LW) ASA.

**Figure 4:**
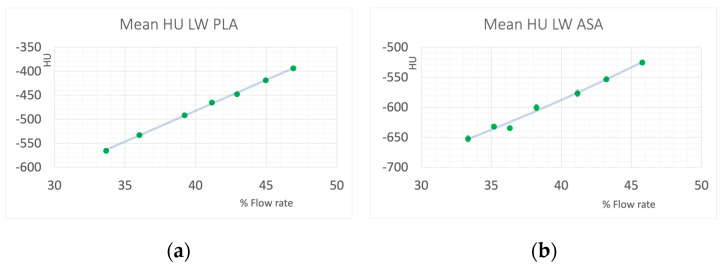
HU values of the samples measured at 120 kV. (**a**): LW PLA, and (**b**): LW ASA.

Figure 3 shows the relation of flow rate and printing temperature resulting in accurate dimensions of the print samples for LW PLA and LW ABS, and the achieved mass densities of cylinders printed with the respective combinations of flow rate and temperature. A wide range of mass densities could be covered, whereas LW PLA was easier to print and yielded more consistent results. Figure 4 shows the resulting HU values in the CT scan. With the foaming materials, HU values from −652 to −590 could be covered with LW ASA, and from −566 to −394 using LW PLA. Trying to print beyond these limits by adjusting temperature in a wider range resulted in printing failures.

### 3.2. Reduced Flow Rates in Regular Printing Materials

With regular (modified) PLA, Nylon, and ABS filaments, the mass density in the printout could be reduced to about 75 to 80 percent before the structural integrity of the printed objects breaks down. This typically happens in the inner parts of the printed objects, while the outer walls still look intact.

**Figure 5:**
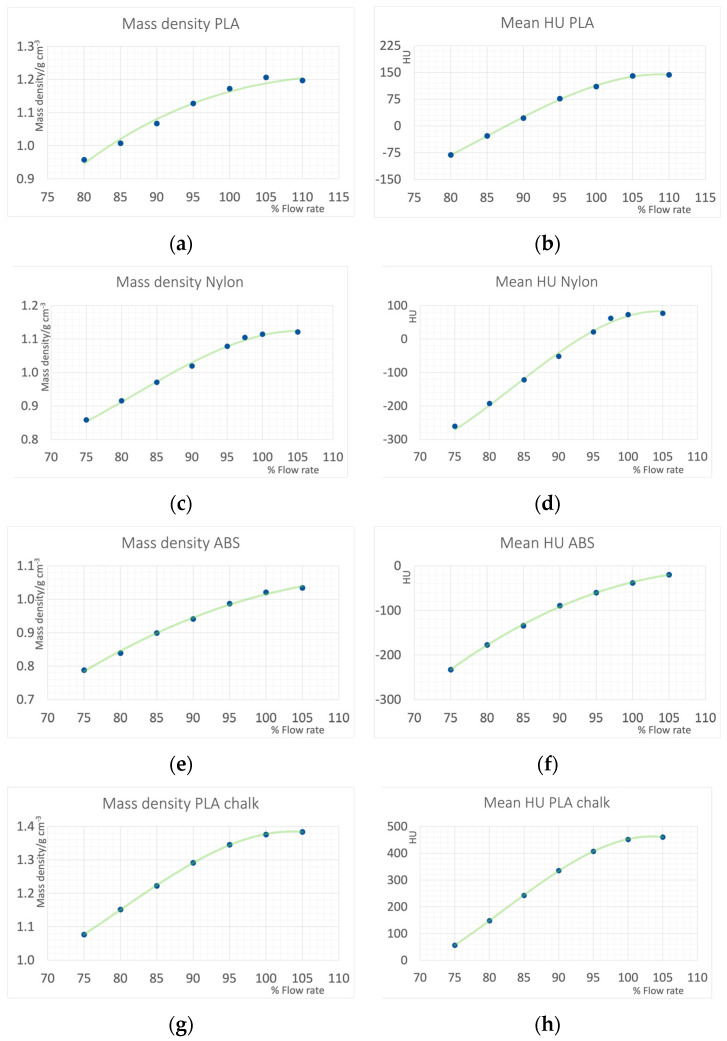
Achieved mass densities and HU values measured on 120 kV CT scan of the samples printed with various flow rates with standard filaments: (**a**,**b**): PLA (modified, tough), (**c**,**d**): Nylon (Polyamide), (**e**,**f**). ABS (modified for low warping and thus easier printing and low odor), and (**g**,**h**): a PLA based filament doped with calcium carbonate (PLA chalk).

The resulting mass densities and CT Hounsfield numbers are shown in Figure 5. For PLA data from 80 to 110% extrusion is shown, and in the other materials from 75% to 105%. Extrusion rates above 100% are commonly used and necessary to reach the maximum density. This is seen in all materials. Increasing the extrusion rate from 100% to 105% results in density and HU increases. HU values of the water filled cylinders were 1.57 ± 1.51, and in the gelatin were 14.40 ± 1.04.

#### Noise Levels Measured in Density Reduced Samples

The average noise level measured in the printed cylinders can serve as an indicator of introduced inhomogeneities, or structural noise, if compared to noise measured in homogeneous samples. Eight water-filled cylinders were placed between the polymer cylinders throughout the phantom to serve as a standard for HU accuracy and quantum noise. Average noise levels measured in the water-filled cylinders as standard deviation of voxel values were 7.27 ± 0.77.

**Figure 6:**
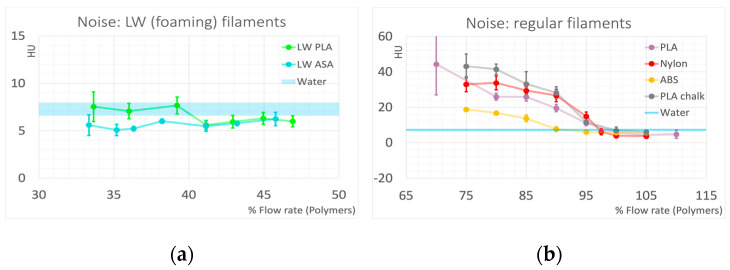
Noise levels measured in the printed cylinders compared to the noise in water measured in the water filled cylinders. (**a**): Light-weight (foaming) filaments; (**b**): regular printing filaments. Error bars correspond to one standard deviation; for water, this is indicated by the thickness of the line.

**Figure 7:**
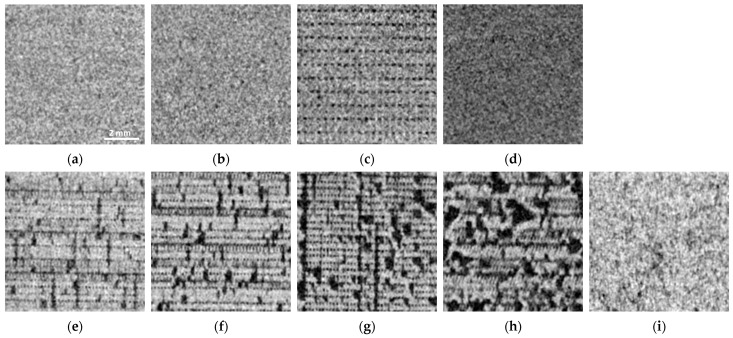
Micro-CT images of the samples: (**a**–**d**): Light-weight (foaming) polymers: (**a**–**c**): LW PLA with flow rates of 46.9% (printed with 260 °C), 41.1% (245 °C) and 33.5% (230 °C), respectively. (**d**): LW ASA (40%, 243 °C). (**e**–**i**): Micro-CT images of samples printed with regular polymer filaments. (**e**–**h**): PLA printed with 80%, 70%, 60%, and 50%, respectively. (**i**): ABS, 75% flow rate. Dimension of all images: 8 by 8 mm.

In Figure 6, the noise levels measured in the polymer cylinders are compared to the noise determined in water. In both foaming (light-weight) polymers, LW PLA and LW ASA, noise levels are comparable to noise in water for all printing densities, indicating inhomogeneities introduced by the foaming process were much smaller than the spatial resolution of the CT. This can be verified in the Micro-CT scans (Figure 7a–d). Visible air inclusions can only be seen in the Micro-CT scan with 35.4^3^ µm^3^ voxel dimension in LW PLA printed with the lowest achievable mass density (33.5%, Figure 7c).

This behavior is different when controlled underextrusion by reducing flow rate in regular thermoplastic polymer filaments is applied. However, the rate of underextrusion resulting in quite homogeneous prints strongly depends on the polymer. ABS best tolerates the reduction in density by reducing the flow rate. This is seen in the noise measurements (Figure 6b), where noise in ABS is in the range of water until a density reduction to 90%, and then only increases at a rate much less than for the other polymers. The PLA used in this study exhibited the highest structural noise if underextruded, indicating poorest structural integrity. The different behavior (compared to ABS) is also evident on the Micro-CT images. Figure 7e–h show the internal structure of underfilled PLA. Since 80% (Figure 7e) can be regarded as the lower acceptable limit, the lower densities have been omitted in the data evaluation of this work, because the structural integrity of the prints is no longer guaranteed (also compare Figure 5a,b). If underextruded PLA is used to simulate liquid water in CT phantoms for quality control or calibration purposes, a filling ratio of approximately 85.7% would be necessary, resulting in approximately three times the noise level seen in liquid water.

Nylon, exhibiting a HU value of 77 HU when printed with maximum density, tolerates underextrusion better than PLA, allowing its use for a wider range of tissue mimicking materials. This makes Nylon a possible choice to simulate soft and adipose tissues in the range of −200 to 70 HU. However, in the negative HU range, ABS is more suitable with regard to low structural noise. A 90% flow rate, associated with minimal or even undiscernible increase in noise compared to water, corresponds to −89 HU, where typically many adipose tissues are found.

Using PLA chalk, the calcium-filled PLA employed in this study, a standard material that mimics average whole bone with 500 HU at 120 kVp with minimal structural noise introduction, can easily be produced, as only minimal underfilling is required. When aiming to replicate average soft bone (350 HU), the resulting inhomogeneities fall within an acceptable range. PLA chalk exhibits an almost optimal energy dependence for simulating softer bone, thanks to its elemental calcium content.

## 4. Discussion

Dedicated low density filaments designed for the modelling hobby to allow for the printing of structurally stable light-weight parts can serve as phantom materials mimicking low density tissues like lungs. While some are easily printable with very stable results—as the two presented in this work—others (not presented) were difficult to print and results (e.g., densities achieved) varied between printing runs. From the regular filaments, ABS best tolerated a density reduction by simply reducing the flow rate, but all filaments yielded usable results when the density was reduced by up to approximately 20%. Lower densities can be achieved by combining reduced flow rates with infill patterns. If these patterns are chosen appropriately [49], and the infill percentage is not too low, little structural noise addition is anticipated.

Printing parameters have to be carefully selected when using underfilling. The resulting densities are not only dependent on the flow rate selected, but also on many other parameters, such as printing speed, layer height, temperature fluctuations, retraction settings, or nozzle dimensions, to name a few [32,35]. Care must be taken if printing parameters like printing speeds are varied, e.g., when outer walls are printed with lower speeds compared to the inner filling. It is then very likely that this results in higher densities in the outer walls as compared to the filling.

Okkalidis and coworkers [28,34] used a pixel-by-pixel (PbP method) approach to vary the deposited material, following the same idea as the variation of the extrusion rate in this work using PLA as printing material. This approach allows for printing a phantom with the possibility of adjusting X-ray attenuation voxel-wise directly from a CT image. However, absolute values of HU depending on underfilling are difficult to compare. Also, resulting HU values in a phantom mimicking brain tissue varied between pixels considerably (−40 to +89) [34], indicating poor inhomogeneity, fostering the result that below approximately a density reduction of >20 percent, the structural integrity of the printed material breaks down. In the PbP approach, since flow per travel distance of the extruder varies continuously, oozing of the material is an additional complication. A discrepancy in resulting densities between using the PbP and infill pattern approach with variable infill density hampers the interpretation and comparison with this work. Nevertheless, a non-linearity for small reductions as also seen in Figure 5 was found in the PbP approach.

Madamesila and coworkers [48] applied infill patterns to reduce average mass density to simulate lung tissues. They report a linear reduction in relative electron density with infill rate using a polystyrene filament. They were able to reproduce appropriate HU (approximately −800 for low density lung tissue, and approximately −480 for high density lung tissue); however, with high standard deviation due to the inhomogeneities introduced by the infill pattern.

Mei and coworkers demonstrated that using printing nozzles with smaller orifice allowed controlled underfilling using a regular PLA filament without introducing macroscopic inhomogeneities due to loosing structural integrity in the volumetric network of the printed sample for lower flow rates as could be achieved in this work [52]. However, this increases printing times. Mei and coworkers report a printing time of 16 h for a calibration cylinder with 10 cm diameter and 1 cm height using a 0.25 mm brass nozzle. They were able to produce samples with a HU range from −867 to 115 measured at 100 kV.

Ozsoykal and Yurt used a different approach to evaluate foaming filaments as density-adjustable soft tissue equivalent phantom materials [53]. The foaming filament used was also different and, contrary to the ones used in this work, exhibited elevated foaming, resulting in lower mass density with increasing temperature. Rather than calibrating the flow rate to the printing temperature to result in correct printing dimensions, Ozsoykal and Yurt used a range of flow rates at the same temperature, and were able to cover a very wide range of densities and, thus, HU values. However, using the same flow rate at different temperatures results in different HU values (e.g., 70% flow rate at 200 °C: −230 HU, at 220 °C −176 HU, 230 °C: −195 HU) indicating the material is extremely sensitive to both controllable und uncontrollable parameters. Nevertheless, this material looks very promising, but extremely well-controlled printing conditions seem to be necessary to result in predictable results. As Ozsoykal and Yurt also state, this filament is very sensitive to heat transfer, printing temperature fluctuations, and accuracy, but also flow rate, print speed, cooling, horizontal cross-section influencing cooling, or temperature gradients, that may all influence the resulting density and, thus, X-ray attenuation.

Tong and coworkers suggested a backfilling approach to overcome the issues of collapsing spatial structure in underfilled areas [49]. In this approach, an additional polymer layer is extruded into an existing background layer (“backfilled”) with adjustable extrusion rate allowing a dynamic range of 200 HU that can be used to create structures and contrasts, whereas the variation of infill percentages from 40 to 100% applying a rectilinear infill pattern with nine infill angles was found to also have a good uniformity, but still larger inhomogeneity than commercial phantoms.

In this study, a limited selection of materials was evaluated, showing that different polymers respond differently to underfilling. The PLA and ABS used in this work were filaments with modified chemical compositions. Thus, the results may vary when other brands or products are used. Future work will aim at adding more materials, and combing different methods to control density in one print to enable the production of anthropomorphic phantoms with a wide range of X-ray attenuations.

A promising strategy for density control in future work could involve integrating different approaches like infill patterns, underextrusion, and backfilling. Additionally, optimizing the density with a wide dynamic range can be achieved by combining various density control strategies and a variation of printing materials through a backfilling technique. Specifically, a dual approach can be implemented: utilizing base layers printed with expanding low-density polymers at reduced flow rates, followed by backfilling with standard-density polymers for phantoms requiring low to very low attenuation, or high attenuation filaments for phantoms containing soft tissue and bone tissue.

## 5. Conclusions

In order to replicate the X-ray interaction properties of tissues or standard materials such as liquid water in radiographic phantoms, many 3D printing materials exhibit a slightly elevated mass density. Adjusting the print density downwards by a few to several percentage points, tailored to the specific polymer used, can achieve near-perfect tissue simulation across a broad spectrum of tissue types. Intentional underfilling of conventional thermoplastic printing polymers offers a viable strategy to address this discrepancy. For applications requiring materials with exceptionally low density and minimal inhomogeneities beyond the typical resolution of imaging systems, utilizing foaming polymers compatible with FDM printing presents a promising solution.

## Figures and Tables

**Table 1:** FDM materials and printer used for production of test samples.

Polymer Type	Filament Name and Manufacturer	Printer Used
**PLA**, Polylactic acid, modified *	EcoPLA tough transparent, 3DJake, Paldau, Austria	Anycubic Vyper
**Nylon**, Polyamide	Nylon transparent; Ultimaker BV, Utrecht, The Netherlands	Ultimaker S3
**ABS**, Acrylonitrile butadiene styrene, modified *	TitanX natural, Formfutura BV, Nijmegen, The Netherlands	Anycubic Vyper
**PLA chalk**, Polylactic acid with chalk powder	PLA Mineral natural; Fiberlogy SA, Brzezie, Poland	Ultimaker S3
Light-weight (**LW**) **ABS**, Acrylonitrile butadiene styrene, modified, with foaming agent	LW-ASA natural, colorFabb BV, Belfeld, The Netherlands	Ultimaker S3
Light-weight (**LW**) **PLA**, Polylactic acid, modified, with foaming agent	PLA LW natural, Recreus, Recreus Industries, S.L., Elda, Spain	Flsun V400

* modified to result in improved toughness (PLA) or print with better accuracy, less warping and less emission of volatile organic compounds (VOCs) during printing (ABS).

## Data Availability

Data are contained within the article.

## References

[1] Tino R., Yeo A., Leary M., Brandt M., Kron T. (2019). A Systematic Review on 3D-Printed Imaging and Dosimetry Phantoms in Radiation Therapy. Technol. Cancer Res. Treat..

[2] Filippou V., Tsoumpas C. (2018). Recent advances on the development of phantoms using 3D printing for imaging with CT, MRI, PET, SPECT, and ultrasound. Med. Phys..

[3] Suleiman O.H., Stern S.H., Spelic D.C. (1999). Patient dosimetry activities in the United States: The nationwide evaluation of X-ray trends (NEXT) and tissue dose handbooks. Appl. Radiat. Isot..

[4] IAEA (2007). Dosimetry in Diagnostic Radiology: An International Code of Practice.

[5] Smet M.H., Breysem L., Mussen E., Bosmans H., Marshall N.W., Cockmartin L. (2018). Visual grading analysis of digital neonatal chest phantom X-ray images: Impact of detector type, dose and image processing on image quality. Eur. Radiol..

[6] Irnstorfer N., Unger E., Hojreh A., Homolka P. (2019). An anthropomorphic phantom representing a prematurely born neonate for digital x-ray imaging using 3D printing: Proof of concept and comparison of image quality from different systems. Sci. Rep..

[7] Hatamikia S., Kronreif G., Unger A., Oberoi G., Jaksa L., Unger E., Koschitz S., Gulyas I., Irnstorfer N., Buschmann M. (2022). 3D printed patient-specific thorax phantom with realistic heterogenous bone radiopacity using filament printer technology. Z. Med. Phys..

[8] Carton A.-K., Bakic P., Ullberg C., Derand H., Maidment A.D.A. (2011). Development of a physical 3D anthropomorphic breast phantom. Med. Phys..

[9] Kiarashi N., Nolte A.C., Sturgeon G.M., Segars W.P., Ghate S.V., Nolte L.W., Samei E., Lo J.Y. (2015). Development of realistic physical breast phantoms matched to virtual breast phantoms based on human subject data. Med. Phys..

[10] Schopphoven S., Cavael P., Bock K., Fiebich M., Mader U. (2019). Breast phantoms for 2D digital mammography with realistic anatomical structures and attenuation characteristics based on clinical images using 3D printing. Phys. Med. Biol..

[11] Hazelaar C., van Eijnatten M., Dahele M., Wolff J., Forouzanfar T., Slotman B., Verbakel W. (2018). Using 3D printing techniques to create an anthropomorphic thorax phantom for medical imaging purposes. Med. Phys..

[12] Hernandez-Giron I., den Harder J.M., Streekstra G.J., Geleijns J., Veldkamp W.J.H. (2019). Development of a 3D printed anthropomorphic lung phantom for image quality assessment in CT. Phys. Med..

[13] Homolka P., Figl M., Wartak A., Glanzer M., Dunkelmeyer M., Hojreh A., Hummel J. (2017). Design of a head phantom produced on a 3D rapid prototyping printer and comparison with a RANDO and 3M lucite head phantom in eye dosimetry applications. Phys. Med. Biol..

[14] Leary M., Kron T., Keller C., Franich R., Lonski P., Subic A., Brandt M. (2015). Additive manufacture of custom radiation dosimetry phantoms: An automated method compatible with commercial polymer 3D printers. Mater. Des..

[15] Carver D.E., Kost S.D., Fraser N.D., Segars W.P., Pickens D.R., Price R.R., Stabin M.G. (2017). Realistic phantoms to characterize dosimetry in pediatric CT. Pediatr. Radiol..

[16] Bolch W., Lee C., Wayson M., Johnson P. (2010). Hybrid computational phantoms for medical dose reconstruction. Radiat. Environ. Biophys..

[17] Petoussi-Henss N., Bolch W.E., Eckerman K.F., Endo A., Hertel N., Hunt J., Menzel H.G., Pelliccioni M., Schlattl H., Zankl M. (2014). ICRP Publication 116—The first ICRP/ICRU application of the male and female adult reference computational phantoms. Phys. Med. Biol..

[18] Segars W.P., Sturgeon G., Mendonca S., Grimes J., Tsui B.M.W. (2010). 4D XCAT phantom for multimodality imaging research. Med. Phys..

[19] Abadi E.A.-O., Segars W.P., Tsui B.A.-O., Kinahan P.A.-O., Bottenus N.A.-O., Frangi A.A.-O.X., Maidment A., Lo J.A.-O., Samei E. (2020). Virtual clinical trials in medical imaging: A review. J. Med. Imaging.

[20] Kalender W.A., Suess C., Faust U. (1988). Polyethylene-based water- and bone-equivalent materials for calibration phantoms in quantitative computed tomography. Biomed. Technol..

[21] Leithner R., Knogler T., Homolka P. (2013). Development and production of a prototype iodine contrast phantom for CEDEM. Phys. Med. Biol..

[22] Euler A., Solomon J., Mazurowski M.A., Samei E., Nelson R.C. (2019). How accurate and precise are CT based measurements of iodine concentration? A comparison of the minimum detectable concentration difference among single source and dual source dual energy CT in a phantom study. Eur. Radiol..

[23] Krauss B., Grant K.L., Schmidt B.T., Flohr T.G. (2015). The importance of spectral separation: An assessment of dual-energy spectral separation for quantitative ability and dose efficiency. Investig. Radiol..

[24] Szucs-Farkas Z., Verdun F.R., von Allmen G., Mini R.L., Vock P. (2008). Effect of X-ray Tube Parameters, Iodine Concentration, and Patient Size on Image Quality in Pulmonary Computed Tomography Angiography: A Chest-Phantom-Study. Investig. Radiol..

[25] Wen G., Markey M.K., Park S. (2017). Model observer design for multi-signal detection in the presence of anatomical noise. Phys. Med. Biol..

[26] Verdun F.R., Racine D., Ott J.G., Tapiovaara M.J., Toroi P., Bochud F.O., Veldkamp W.J., Schegerer A., Bouwman R.W., Giron I.H. (2015). Image quality in CT: From physical measurements to model observers. Phys. Med..

[27] Barrett H.H., Yao J., Rolland J.P., Myers K.J. (1993). Model observers for assessment of image quality. Proc. Natl. Acad. Sci. USA.

[28] Okkalidis N. (2018). A novel 3D printing method for accurate anatomy replication in patient-specific phantoms. Med. Phys..

[29] Solomon J., Bochud F., Samei E. Design of anthropomorphic textured phantoms for CT performance evaluation. Proceedings of the Medical Imaging 2014: Physics of Medical Imaging, 90331U.

[30] Solomon J., Samei E. (2014). Quantum noise properties of CT images with anatomical textured backgrounds across reconstruction algorithms: FBP and SAFIRE. Med. Phys..

[31] Solomon J., Ba A., Bochud F., Samei E. (2016). Comparison of low-contrast detectability between two CT reconstruction algorithms using voxel-based 3D printed textured phantoms. Med. Phys..

[32] Ma X., Buschmann M., Unger E., Homolka P. (2021). Classification of X-Ray Attenuation Properties of Additive Manufacturing and 3D Printing Materials Using Computed Tomography from 70 to 140 kVp. Front. Bioeng. Biotechnol..

[33] Silvestro E., Betts K.N., Francavilla M.L., Andronikou S., Sze R.W. (2020). Imaging Properties of Additive Manufactured (3D Printed) Materials for Potential Use for Phantom Models. J. Digit. Imaging.

[34] Okkalidis N., Marinakis G. (2020). Technical Note: Accurate replication of soft and bone tissues with 3D printing. Med. Phys..

[35] Craft D.F., Kry S.F., Balter P., Salehpour M., Woodward W., Howell R.M. (2018). Material matters: Analysis of density uncertainty in 3D printing and its consequences for radiation oncology. Med. Phys..

[36] Dancewicz O.L., Sylvander S.R., Markwell T.S., Crowe S.B., Trapp J.V. (2017). Radiological properties of 3D printed materials in kilovoltage and megavoltage photon beams. Phys. Med..

[37] Alssabbagh M., Tajuddin A.A., bin Abdul Manap M., Zainon R. (2017). Evaluation of nine 3D printing materials as tissue equivalent materials in terms of mass attenuation coefficient and mass density. Int. J. Adv. Appl. Sci..

[38] Homolka P., Gahleitner A., Prokop M., Nowotny R. (2002). Optimization of the composition of phantom materials for computed tomography. Phys. Med. Biol..

[39] Tan Z., Dini D., Rodriguez y Baena F., Forte A.E. (2018). Composite hydrogel: A high fidelity soft tissue mimic for surgery. Mater. Des..

[40] Ali A., Wahab R., Huynh J., Wake N., Mahoney M. (2020). Imaging properties of 3D printed breast phantoms for lesion localization and Core needle biopsy training. 3D Print Med..

[41] Tejo-Otero A., Lustig-Gainza P., Fenollosa-Artes F., Valls A., Krauel L., Buj-Corral I. (2020). 3D printed soft surgical planning prototype for a biliary tract rhabdomyosarcoma. J. Mech. Behav. Biomed. Mater..

[42] Tan Z., Parisi C., Di Silvio L., Dini D., Forte A.E. (2017). Cryogenic 3D Printing of Super Soft Hydrogels. Sci. Rep..

[43] Lappchen T., Meier L.P., Furstner M., Prenosil G.A., Krause T., Rominger A., Klaeser B., Hentschel M. (2020). 3D printing of radioactive phantoms for nuclear medicine imaging. EJNMMI Phys..

[44] Yunker B.E., Holmgren A., Stupic K.F., Wagner J.L., Huddle S., Shandas R., Weir R.F., Keenan K.E., Garboczi E., Russek S.E. (2020). Characterization of 3-Dimensional Printing and Casting Materials for use in Computed Tomography and X-ray Imaging Phantoms. J. Res. Natl. Inst. Stand. Technol..

[45] Ma X., Figl M., Unger E., Buschmann M., Homolka P. (2022). X-ray attenuation of bone, soft and adipose tissue in CT from 70 to 140 kV and comparison with 3D printable additive manufacturing materials. Sci. Rep..

[46] Ivanov D., Bliznakova K., Buliev I., Popov P., Mettivier G., Russo P., Di Lillo F., Sarno A., Vignero J., Bosmans H. (2018). Suitability of low density materials for 3D printing of physical breast phantoms. Phys. Med. Biol..

[47] Cockmartin L., Marshall N.W., Zhang G., Lemmens K., Shaheen E., Van Ongeval C., Fredenberg E., Dance D.R., Salvagnini E., Michielsen K. (2017). Design and application of a structured phantom for detection performance comparison between breast tomosynthesis and digital mammography. Phys. Med. Biol..

[48] Madamesila J., McGeachy P., Villarreal Barajas J.E., Khan R. (2016). Characterizing 3D printing in the fabrication of variable density phantoms for quality assurance of radiotherapy. Phys. Med..

[49] Tong H., Pegues H., Samei E., Lo J.Y., Wiley B.J. (2022). Technical note: Controlling the attenuation of 3D-printed physical phantoms for computed tomography with a single material. Med. Phys..

[50] Mille M.M., Griffin K.T., Maass-Moreno R., Lee C. (2020). Fabrication of a pediatric torso phantom with multiple tissues represented using a dual nozzle thermoplastic 3D printer. J. Appl. Clin. Med. Phys..

[51] Khalil M.M. (2017). Performance characteristics of the Inveon micro-CT scanner in small animal imaging. Biomed. Phys. Eng. Express.

[52] Mei K., Geagan M., Roshkovan L., Litt H.I., Gang G.J., Shapira N., Stayman J.W., Noel P.B. (2022). Three-dimensional printing of patient-specific lung phantoms for CT imaging: Emulating lung tissue with accurate attenuation profiles and textures. Med. Phys..

[53] Ozsoykal I., Yurt A. (2024). Introduction of a Novel Technique in Density-Adjusted 3D Printing for the Manufacture of Soft-Tissue-Equivalent Radiological Phantoms. Appl. Sci..

